# Music training with Démos program positively influences cognitive functions in children from low socio-economic backgrounds

**DOI:** 10.1371/journal.pone.0216874

**Published:** 2019-05-16

**Authors:** Mylène Barbaroux, Eva Dittinger, Mireille Besson

**Affiliations:** 1 CNRS & Aix-Marseille University, Laboratoire de Neurosciences Cognitives (LNC, UMR 7291), Marseille, France; 2 CNRS & Aix-Marseille University, Laboratoire Parole et Langage (LPL, UMR 7309), Aix-en-Provence, France; 3 Brain and Language Research Institute (BLRI), Aix-en-Provence, France; University of California, San Francisco, UNITED STATES

## Abstract

This study aimed at evaluating the impact of a classic music training program (Démos) on several aspects of the cognitive development of children from low socio-economic backgrounds. We were specifically interested in general intelligence, phonological awareness and reading abilities, and in other cognitive abilities that may be improved by music training such as auditory and visual attention, working and short-term memory and visuomotor precision. We used a longitudinal approach with children presented with standardized tests before the start and after 18 months of music training. To test for pre-to-post training improvements while discarding maturation and developmental effects, raw scores for each child and for each test were normalized relative to their age group. Results showed that Démos music training improved musicality scores, total IQ and Symbol Search scores as well as concentration abilities and reading precision. In line with previous results, these findings demonstrate the positive impact of an ecologically-valid music training program on the cognitive development of children from low socio-economic backgrounds and strongly encourage the broader implementation of such programs in disadvantaged school-settings.

## Introduction

Music training programs for children and adolescents from low socio-economic status (SES), defined based on parental income and education, have flourished in many countries since the fifties, not only to promote music education but also to increase quality of life, general education and the cohesion of social groups. For instance, the Yehudi Menuhin school was founded in England by the well-known violinist in 1963; the El Sistema project was created in Venezuela in 1975 by the musician and economist José Antonio Abreu and was later extended to Canada, Europe -England, France, Greece, Portugal- and more than 80 El Sistema-inspired programs are active throughout the United States. More recently, the Harmony project was launched in Los Angeles suburbs in the years 2000 to improve education for youth from low-income communities. Similarly, the Démos project (“Dispositif d’éducation musicale et orchestrale à vocation sociale”, Musical and orchestral education with social vocation: http://projetdemos.fr/qu-est-ce-que-demos.aspx) that is pivotal to the research presented here, was initiated by the Paris Philharmonie in 2010, with the objective to promote the cognitive development and the social integration of children from disadvantaged backgrounds through access to culture and free classic music training. However, the societal impact of these music programs has not often been measured using a scientific approach. Our main objective here was to evaluate the impact of the Démos program on the cognitive development of children from disadvantaged backgrounds using a longitudinal approach. To this end, we tested the children before the start of the music intervention and 18 months later (test–music training–retest procedure) using standard tests of general intelligence (Intelligence Quotient, IQ), of phonological awareness and reading abilities as well as of other cognitive functions, including auditory and visual attention, working and short-term memory and sensorimotor precision.

The issue of whether music training “makes you smarter” is highly controversial. There is clear evidence that music training in children is positively correlated to higher cognitive functioning. For instance, in several studies using the full or reduced (four tests) versions of the Wechsler Intelligence Scale for Children (WISC), Schellenberg and collaborators showed higher IQ scores in musically-trained children than in their untrained counterparts [1–3]. How to interpret this association between music training and IQ is less clear-cut. Schellenberg [1] pointed out that while music training may be the cause of improved cognitive functioning, children with higher cognitive functioning are possibly more likely to engage in (and to pursue) the demanding task of learning to play music than children with lower cognitive abilities. To directly address this issue, Schellenberg [4] conducted the first well-controlled longitudinal study with a large group of 6-year old children (N = 132) trained either in music, drama or no specific training. After one year of training, increase in IQ scores was significantly larger in the music group than in the control groups (6.1 vs 3.9 IQ points), thereby providing causal evidence for a positive impact of music training on general intelligence in children from middle to high socio-economic backgrounds. More recently, Sala and Gobet [5] also concluded from the results of a meta-analysis that music training had small but significant beneficial effects on general intelligence. To our knowledge, however, no study has yet focused on socially disadvantaged children. Thus, our first objective was to determine whether music training as provided by the Démos program would also improve measures of general intelligence in children from low socio-economic background.

The second objective was to evaluate the impact of the Démos music program on phonological awareness and reading skills that are frequently impaired in children from low income families [6–8]. While there is evidence that music training improves phonological skills in typically developing children [9,10] (see also results of meta-analysis [11] as well as studies in children with dyslexia [10,12]), the evidence for an impact of music training on reading skills is more controversial [11,13].

To evaluate the impact of the Harmony project mentioned above, Kraus and colleagues [14,15] followed up a group of 6–9 year children from low SES, from the gang reduction zones in Los Angeles, for two years. Results showed a positive correlation between reading fluency (tested using word and non-word reading tests) and the amount of engagement in the Harmony music program (measured as the percentage of attendance and the level of participation in music classes [14]). Importantly, while age-normed reading scores were enhanced in children that were more-engaged in music classes, they tended to deteriorate over time in children that were less-engaged, possibly reflecting the negative consequences of living in low socio-economic backgrounds [16–18]. Moreover, children trained with music for one year outperformed non-trained children in tasks requiring the silent discrimination of written words in a continuous sequence of letters [15]. Again, musically-trained children maintained their reading scores at the age-normed level while they decreased for children in the control group. However, Slater and colleagues [15] reported no significant effects of music training on phonological awareness after one year of music training in the Harmony project.

In sum, results from the Kraus group provided evidence that the Harmony project helped children from low SES to attain and maintain reading levels close to more privileged children but the impact on phonological awareness was less clear-cut. By contrast, very recent results of a longitudinal study by Linnavalli and collaborators [19] showed that music playschool, but not dance lessons (both 45 minutes weekly, 30 times a year), enhanced phonological awareness (e.g., choose the object whose name comprises the combination of phonemes pronounced by the experimenter; suppress or replace a phoneme and say the resulting word) and vocabulary (verbal knowledge) of 5-6-year-old preschool Finnish children. Similarly, Nan and collaborators [20] showed that 6 months of piano training (45 minutes, 3 times per week) improved word discrimination based on consonants compared to reading training in 4-5-year-old Mandarin-speaking children. Importantly, Degé & Schwartzer [21] also showed that music training is possibly as efficient as phonological training and more efficient than sport training (in all cases, 10 min of daily training for 20 weeks), to improve phonological awareness of large phonological units (detecting rhymes and segmenting words into syllables). It was thus of interest to compare these contrastive results to those of the children involved in the Démos music training program.

Finally, previous results also suggest that some aspects of auditory [22] and visual attention [23] (but see [22,24] for negative results) are improved in adult musicians compared to non-musicians. Moreover, results of cross-sectional studies in children have shown that music training is associated with better working and short-term memory as reflected by higher scores at the backward and forward versions of the Digit Span test (i.e., repeat back series of orally presented numbers) in musically-trained compared to untrained children [3,25]. Very recently, Guo and collaborators [26] provided evidence for a causal link between music training and working memory in an interventional study: children quasi-randomly assigned to six weeks of music training increased their backward digit span scores but the forward digit span scores did not differ between the music and control groups. Moreover, Degé and collaborators [27] used a longitudinal approach and showed that children trained in music for two years improved visual and auditory memory (recalling sequences of colors or sounds), while their non-trained counterparts did not. Importantly, children and adolescents from low SES typically show increased difficulties to focus attention [28] together with working memory deficits that can, however, be counteracted by relevant interventions [29]. Based on these findings, our third objective was to use a test-training-retest procedure with children from disadvantaged socio-economic backgrounds to test for the relationship between music training and improvements in these different cognitive abilities, auditory and visual attention, working and short-term memory, as well as in visuomotor precision (strongly needed to make the fine controlled movements required to play a musical instrument). Evaluations of community-based musical interventions are clearly too scarce and often incomplete, and it was of strong societal interest to obtain a global screening of the impact of the Démos music training program on different cognitive and motor abilities.

To these aims, we tested children at two time points, before they started music training and after 18 months of being involved in the Démos program, using standardized tests of several cognitive functions. Moreover, we computed normalized scores both before and after music training so that significant pre-to-post improvements would reveal the influence of the Démos program on children’ cognitive functions rather than the mere influence of maturation and development (children were 18 months older post-than-pre training). Our general prediction was that, rather than showing the typical deterioration of cognitive abilities across the course of development [14–17] music training would increase the level of performance of children from low SES to the age-normed level. Specifically and based on the literature reviewed above, we hypothesized that classic music training would help children to improve their IQ scores [1–4] as well as their reading scores [14,15] and their backward digit span scores [26]. However, predictions were less clear-cut regarding the impact of music training on phonological awareness [15,19,20], auditory attention [22,28,30] and visual attention [22, 23] because contrastive results have been reported in the literature. Finally, based on previous results showing that music training is more beneficial for children with lowest initial performance levels [19, 31]], we also hypothesized that the beneficial effect of music training would be larger for the children with initial lowest scores. In other words, we predicted that music training may, at least to some extent, counteract the deleterious consequences of living in low socio-economic backgrounds. Based on the relatively short duration of music training, we expected these effects to be small but significant.

## Methods

### Participants and procedure

Fifty-four children (24 girls, 30 boys) from two primary schools, located in middle-class areas in downtown Marseille, were involved in the Démos music program. Even if the area is not what would typically be called underprivileged, these schools were chosen by the organizers of the Démos program together with social institutions “Apprentis d’Auteuil” because they are specifically dedicated to the education of children from low socio-economic status. Since our aim was to test the impact of the Démos program implemented by the Paris Philharmonie in two schools, the sample size was constrained by the number of children in each school. Participation to the Démos program was strongly encouraged (only a very few children did not participate or withdraw from the program) and all children in the schools were tested. However, nineteen children could not be re-tested in the second session 18 months later because some children (7) left primary school to go to middle school and 11 children moved to another city/school during the duration of the Démos program. Only one child voluntary asked to stop being involved in the Démos program.

Analyses included the 35 children (18 girls, 17 boys), 7 to 12 years old (2^nd^ to 5^th^ grade in the first session) that were tested both pre and post music training. All children were native French speakers and had normal or corrected vision and normal audition. All children were from low SES families, often single parent families, defined by French government criteria as families with high unemployment levels, low income and social difficulties. This study was verbally approved by the local ethics committee of Aix-Marseille University because it was conducted under the responsibility of the directors and teachers of the two schools in which the “Démos project” was implemented. All parents gave their informed written consent for their children to participate in the study. This study was conducted in agreement with guidelines for the protection of human participants as defined in the declaration of Helsinki. The procedure was carefully explained to the children to ensure that they agreed to participate in individual testing sessions in a quiet classroom of the school. Children were informed that they could stop performing the tests at any time and they were given presents at the end of the pre and post training evaluations to thank them for their participation.

### Music training

Music classes were taught by two professional music teachers specifically trained for interventions in school settings. Children were trained over a 18 months period (except vacation times), twice a week for two hours for a total of 4 hour/week. Small groups of 6–7 children were formed based on the instrument (from the string or wind family) that they had freely chosen. School teachers were involved in the music classes together with the children. The teaching method was inspired from the Suzuki and Kodaly methods that are based on listening, imitation and memorization. Each child learned to recognize the various instruments and to reproduce the sounds and the music played by the teacher on their own instrument. Children were also progressively trained to read musical scores. Finally, additional orchestra classes were given once every 6 weeks for two hours to train the children for a public concert performed with professional musicians from the Opera orchestra at the end of each school year. The total number of training hours over the 18 months period was around 250 hours, without considering the time children spent practicing their instrument at home. At the end of the Post-training session, children were asked how many times and for how long they practiced their instrument at home each week. Most children (around 90%) reported that they did indeed practiced their instrument at home, 2 or 3 times a week for a total duration of 45 min per week.

### Measures

**Fig 1:**
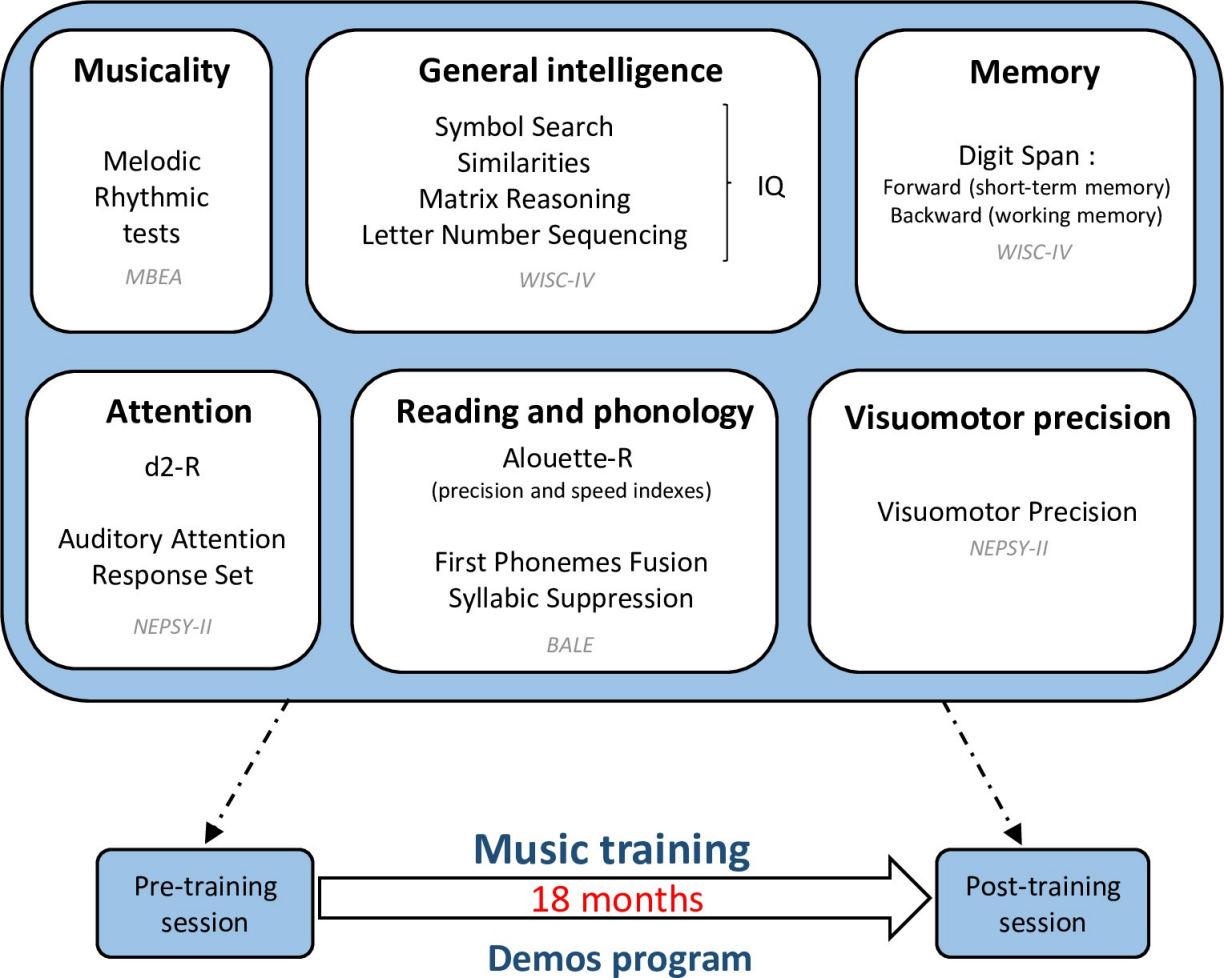
Experimental procedure with the different tests presented before and after 18 months of music training.

The experimental procedure and the different tests are presented on Fig 1.

Two musicality tests adapted from the MBEA (Montreal Battery of Evaluation of Amusia) were used to evaluate musical abilities [32]. Children had to judge whether two successively-presented musical phrases were same or different, based either on melody or on rhythm.

An abbreviated version of the WISC-IV [33] was used to compute IQ scores that comprised one subtest for each four indexes: Symbol Search (processing speed), Similarities (verbal comprehension), Matrix Reasoning (perceptual reasoning), and Letter Number Sequencing (auditory attention and working memory). This short-version has been shown to yield results similar to the full-scale version [34] and provides a precise and reliable estimation of IQ in approximately thirty minutes. The scores for each subtest were normalized and total IQ was computed by adding the four normalized scores, and by converting this score into a standard IQ score using a dedicated conversion table.

*Symbol Search* (WISC-IV*)*: processing speed. On each row, two target symbols are presented on the left side and five symbols are presented on the right side. Children are asked to decide, as fast and as accurately as possible, whether one of the left target symbols is present or not within the five symbols on the right. Each page comprises 15 rows.

*Similarities* (WISC-IV): verbal comprehension. Pairs of words were presented to the child who described how they are alike (e.g. how are red and blue alike? Response: both are colors).

*Matrix Reasoning* (WISC-IV): non-verbal intelligence. A figure is missing from a series of figures and children are asked to choose between five options, the picture that best completes the series.

*Letter Number Sequencing* (WISC-IV): auditory attention and working memory. Series of letters and numbers were read to the child who repeated back the letters in the alphabetical order, and the numbers in numerical order (e.g. 4-A-3-C, response: 3-4-A-C).

The *Digit Span* test (WISC-IV, both forward and backward versions [33]) was administered to further evaluate auditory short-term and working memory. Increasing series of numbers were read to the child who repeated them back in the same order (Forward Digit Span: short-term memory) or in reverse order (Backward Digit Span: working memory).

Auditory and visual attention abilities were evaluated by the *Auditory Attention and Response Set* tests (from the NEPSY-II battery [35]) and by the *d2-R* test [36]. A paper with four colored circles (yellow, red, blue, black) was presented in front of the child who listened to a recorded list of words that included targets and distractors. In the Auditory Attention test, the child pointed to the red circle when hearing the word “red”. In the Response Set test, the child pointed to the red circle when hearing the word “yellow” and vice-versa, and also pointed to the blue circle when hearing the word “blue”. In the d2-R concentration ability test, the child was presented with a sheet of paper where the letters “d” and “p” were surrounded by one to four small strokes. The child was asked to cross out all target letters: the “d” surrounded by two strokes. For each line on the sheet, the child crossed out as many targets as possible in 20 seconds. The test lasted for 5 minutes. The Concentration Capacity index was computed by taking the number of targets examined by the child (within the 20 seconds time-limit) minus the number of missed targets minus the number of incorrectly crossed distractors.

Reading abilities and phonological awareness were assessed using the French standard *Alouette* test (revised version [37]) and the *First Phonemes Fusion* and *Syllabic Suppression tests (*from the BALE battery: Batterie Analytique du Langage Ecrit, analytic battery of written language [38], respectively. The Alouette test measures reading precision and reading speed. The child was asked to read aloud a complex nonsense text within a 3 minutes time limit. Based on the number of words read (M), the number of words correctly read (C), and the reading time (TL), two indexes were computed: the reading precision index [CM = (C/M)x100] and the reading speed index [CTL = (Cx180)/TL]. In the *Phonemes Fusion* test, two words were spoken aloud by the experimenter and the child merged their first phonemes (e.g. “good orange”, response: GO). In the Syllabic Suppression test, bi- or trisyllabic words were spoken aloud by the experimenter and the child supressed either the first, the second or the third syllabe (e.g “suppress the first syllable of the word elephant”, response: LEFANT).

Finally, fine motor skills, speed and precicion of hand-eye visuomotor coordination were measured using the Visuomotor Precision test (NEPSY-II). A sheet of paper picturing a winding road with borders on each side was presented to the child who followed the road with a pencil as fast and as accurately as possible (keeping inside the borders).

All tests were presented in a single session that lasted for about 75 min, with short breaks regularly interspaced during the session and/or on children’ demand.

### Data processing and statistical analyses

All tests are standardized tests (i.e., a large sample of children, representative of the French children population, have been presented with these tests thereby providing a mean reference score for each age range). Moreover, raw scores for each child and for each test were normalized relative to their age group to discard maturation and developmental effects (similar to a passive control group) and these scores were compared between the pre and post training sessions. No active control group was tested (see discussion). Finally, note that standard scores for the musicality tests could not be computed for all children since, to our knowledge, the MBEA only provides normative data for adults [32], 14 to 18 years old adolescents [39] and 6 to 8 years old children [40].

The variable number of children in the different tests (see Table 1) was mainly linked to norms for some age-ranges not being available for some tests so that data from children in these age-groups were not further considered. For instance, for the d2-R, no standardized scores are available below 9 years old. By contrast, for the two BALE tests (Syllabic suppression and Phoneme fusion), no norm is available above 5^th^ grade. As a consequence, data from children above 5^th^ grade in the Post-training session were not included in the analyses (children were included in our sample only when age-norms were available for children both in Pre and Post training sessions). Only in very rare cases children did not want to perform some tests.

**Table 1:** Pre- and post-training results for the different tests.

Test	N	Session	Mean standard score [SD]	z-score [SD]	Cohen’s d	Pre-post comparison (t-test or Wilcoxon test)	Data distribution Shapiro-Wilk: W (p)
***IQ***	30	Pre	81.17 [19.12]	-1.25 [1.27]	0.24	t(29) = -2.45, **p < .02**	.97 (.49)
		Post	85.47 [15.87]	-0.97 [1.06]			.97 (.59)
***Symbol Search***	34	Pre	7.56 [2.88]	-0.81 [0.96]	0.33	t(33) = -2.47, **p < .05**	.97 (.58)
		Post	8.53 [3.05]	-0.49 [1.02]			.95 (.12)
***Similarities***	32	Pre	8.78 [4.29]	-0.41 [1.43]	0.01	t(31) = -0.07, p = .95	.95 (.19)
		Post	8.81 [3.41]	-0.40 [1.14]			.94 (.07)
***Matrix Reasoning***	34	Pre	7.41 [3.56]	-0.86 [1.19]	0.10	t(33) = -0.62, p = .54	.96 (.21)
		Post	7.71 [2.59]	-0.76 [0.86]			.96 (.22)
***Letter-Number Sequencing***	32	Pre	6.84 [3.57]	-1.05 [1.19]	0.15	t(31) = -0.98, p = .33	.97 (.50)
		Post	7.34 [2.90]	-0.89 [0.97]			.97 (.64)
***Digit Span***	34	Pre	7.18 [2.89]	-0.94 [0.96]	0.16	t(33) = -1.02, p = .31	.97 (.43)
		Post	7.62 [2.46]	-0.79 [0.82]			.96 (.28)
***d2-R***	27	Pre	84.63 [10.21]	-1.02 [0.68]	0.85	t(26) = -7.11, **p < .001**	.97 (.55)
		Post	93.48 [10.61]	-0.43 [0.71]			.95 (.24)
***Visuomotor Precision***	32	Pre	9.28 [2.89]	-0.24 [0.96]	0.20	Wilcoxon: Z = 1.30, p = .19	.92 (< .05)
		Post	9.84 [2.58]	-0.05 [0.86]			.96 (.22)
***Auditory Attention***	35	Pre	8.80 [3.14]	-0.40 [1.05]	0.03	Wilcoxon: Z = .30, p = .76	.96 (.24)
		Post	8.89 [3.35]	-0.37 [1.12]			.90 (< .01)
***Response Set***	31	Pre	8.90 [3.08]	-0.37 [1.02]	0.24	Wilcoxon: Z = .88, p = .38	.94 (.06)
		Post	9.58 [2.54]	-0.14 [0.85]			.91 (< .05)
***Syllabic Suppression***	26	Pre	-	-0.55 [1.27]	0.08	Wilcoxon: Z = .22, p = .83	.88 (< .01)
		Post		-0.45 [1.17]			.85 (< .01)
***First Phonemes Fusion***	26	Pre	-	-0.50 [1.27]	0.17	Wilcoxon: Z = .83, p = .41	.88 (< .01)
		Post		-0.30 [1.06]			.92 (< .05)
***Alouette Precision***	33	Pre	-	-1.74 [2.05]	0.19	Wilcoxon: Z = 1.96, **p < .05**	.87 (< .001)
		Post		-1.37 [1.77]			.84 (< .001)
***Alouette Speed***	33	Pre	-	-1.03 [0.90]	0.07	t(33) = -0.64, p = .53	.96 (.24)
		Post		-0.97 [0.89]			.97 (.37)
*Non-standardized data*							
***Melodic test***	35	Pre	57.14 [13.17]	-	0.55	Wilcoxon: Z = 2.22, **p < .05**	.95 (.15)
		Post	64.71 [14.32]				.94 (< .05)
***Rhythmic test***	35	Pre	61.90 [13.42]	-	0.70	t(34) = -4.66, **p < .001**	.95 (.12)
		Post	71.42 [13.82]				.95 (.12)

The normality of data distribution was tested using the Shapiro-Wilk test (W). Student t-tests were used to compare normalized results in the pre vs post training sessions for the tests showing a normal distribution and Wilcoxon tests were used for non-normally distributed dataset. The Statistica software was used for all statistical analyses (Version 12.0 StatSoft, Inc, Tulsa, OK) including the cluster analyses below.

To further investigate whether the observed effects reflected a general trend or whether different trends were present within the group of children, we conducted cluster analyses [41] for the tests showing significant improvements. This allowed us to separate children into three groups: those who showed an improvement (cluster 1), those who showed no change (cluster 2), and those who showed a decrease in performance from pre to post music training (cluster 3). The differences in level of performance pre vs post training were analyzed by interactive partitioning (K-means), minimizing the within-cluster variability and maximizing the between-cluster variability. Then, t-test comparisons of a single value (0) to the mean difference of each cluster were conducted with Bonferroni’s correction (p < .05 divided by 3 tests: significant threshold at .02). Descriptive analyses were also conducted to determine the percentage of children improving in the test(s) showing significant effects of music training.

Finally, simple and multiple linear regression analyses were computed for the tests showing significant pre-post differences to determine which factor(s) contributed to explain the results.

## Results

### Test for the normality of the data distribution

Data distribution was normal for rhythmic musicality, total IQ, Symbol Search, Similarities, Matrix Reasoning, Letter-Number Sequencing, Digit Span, d2-R, and the speed index of the Alouette reading test. Therefore, the Student t-test for normally-distributed dataset was used to compare pre vs post results. By contrast, data were not normally distributed pre and/or post training for melodic musicality, Auditory Attention, Response Set, Syllabic Suppression, First Phonemes Fusion, the precision index of the Alouette reading test and for Visuomotor Precision. In these cases, the Wilcoxon test for non-normally-distributed dataset was used to compare pre vs post results (see Table 1).

Results at the different tests listed in the first column. N is the number of children who performed the tests both in the pre and in the post-training sessions. Mean standard scores and z-scores are presented together with the effect size (Cohen’s d) and the results of statistical analyses using t-tests or Wilcoxon tests depending upon the normality of the data distribution, as reported in the last column.

### Pre-post comparison

**Fig 2:**
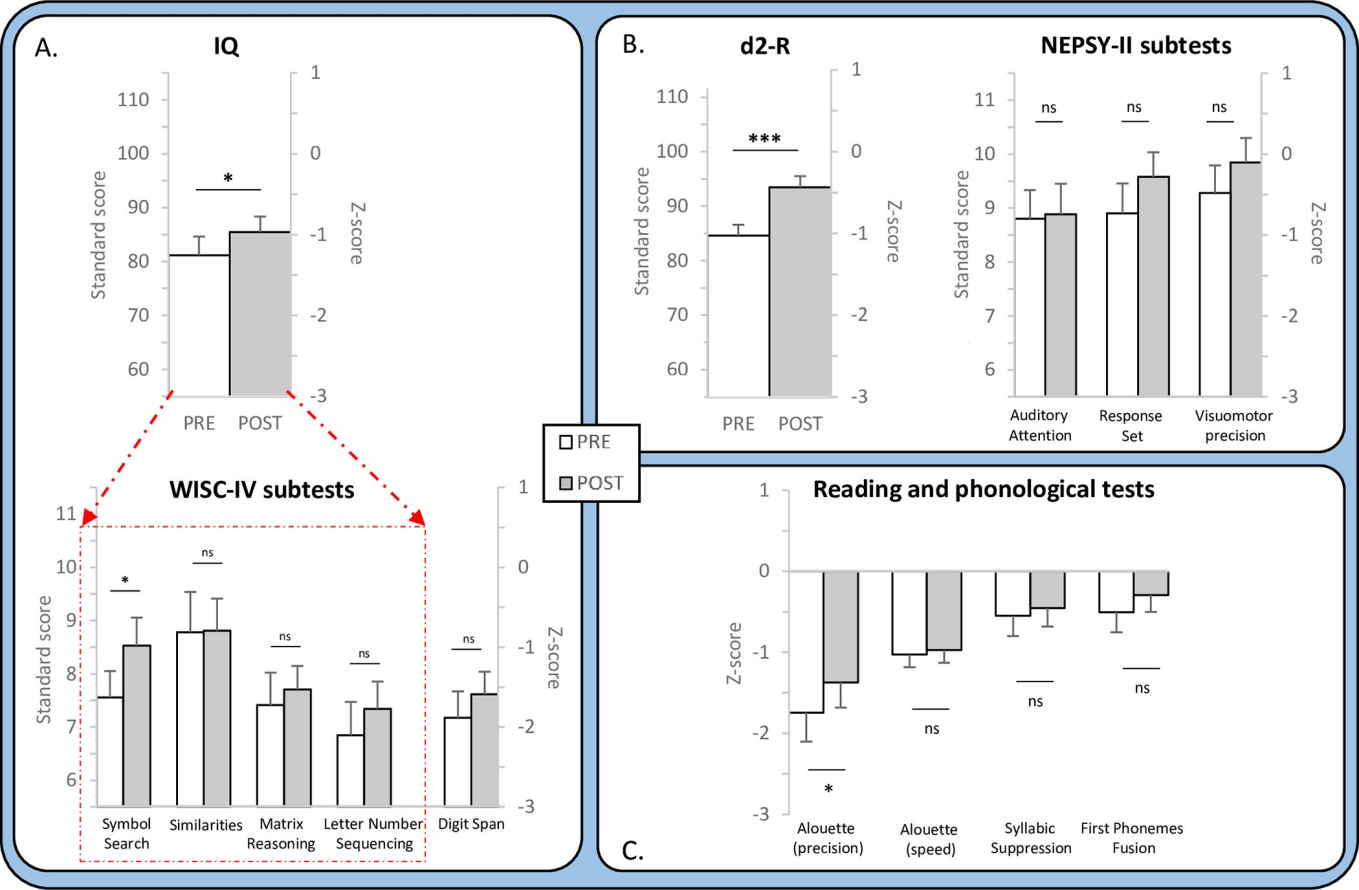
Pre- and post-training results for all tests. A. Results for total Intelligence Quotient (IQ) score, for the four subtests of the reduced version of WISC-IV and for the digit span test are compared in the pre- and post-music training sessions. B. Results at the d2-R and NEPSY-II subtests are compared in the pre- and post-music training sessions. For both A and B, standard scores are indicated on the left ordinate and z-scores on the right ordinate. C. Results at the Alouette reading test and at the phonological tests are compared in the pre- and post-music training sessions. Z-scores are indicated on the left ordinate. Significant pre vs post differences with *: p < .05; ***: p < .001; ns: not significant. Error bars are standard errors of mean (SEM).

**Fig 3:**
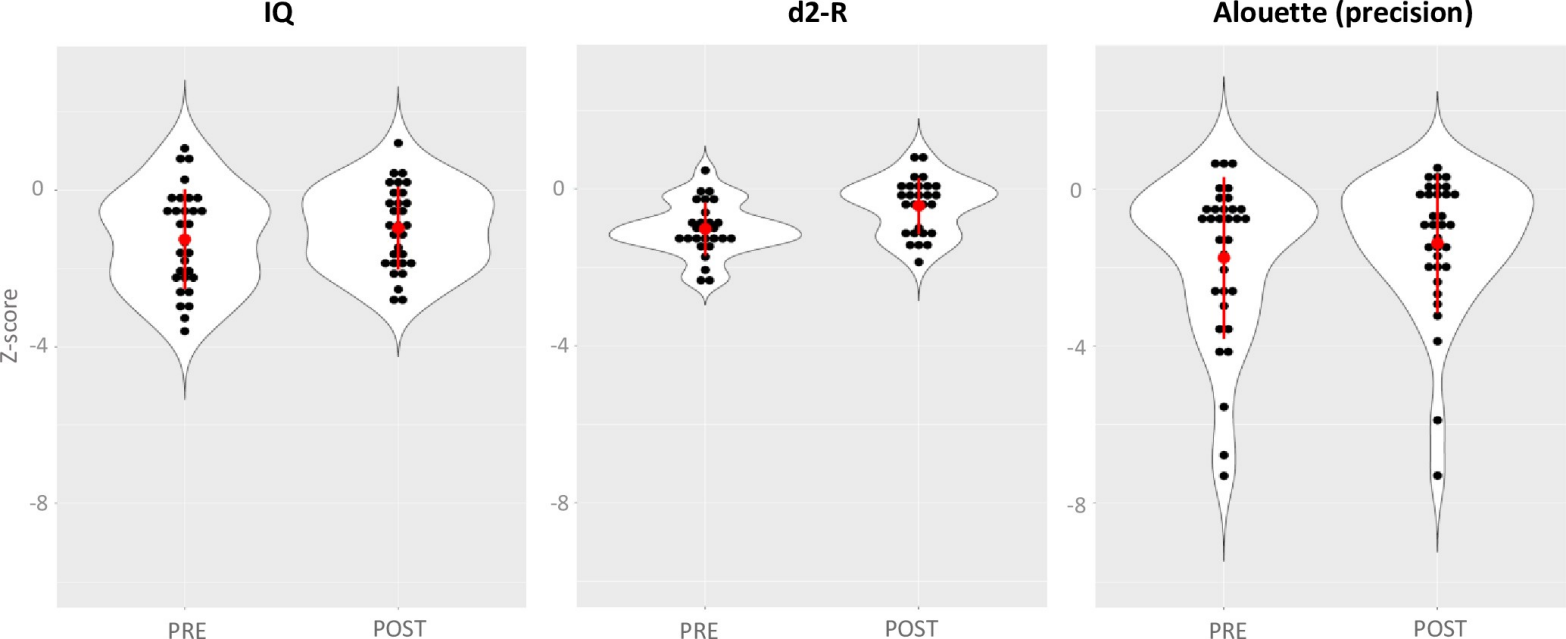
Violin plots for each test showing significant improvements from pre to post-music training. Violin plots (*R* Development Core Team, 2018) show the shape of the distribution. Each black dot represents one child, the red dot corresponds to the mean and the red line to the standard deviation (SD). Z-scores are indicated on the ordinate.

Results at the different tests are presented in Table 1 and illustrated on Fig 2 (averaged data) and on Fig 3 (individual data). They showed a significant improvement in musicality scores with music training, as reflected by higher percentage of correct responses post- than pre-training in both the melodic (Pre: 57.14, Post: 64.71; Z = 2.22, p < .05; Cohen’s d = 0.55) and the rhythmic tests (Pre: 61.90, Post: 71.42; t(34) = -4.66, p < .001; Cohen’s d = 0.70).

Results also showed a significant improvement of total IQ from pre (81.17) to post music training (85.47; t(29) = -2.45, p = .02; Cohen’s d = 0.24) when considering the four subtests of the WISC-IV abbreviated version. Considering each subtest separately, the pre to post improvement was only significant in the Symbol Search test (pre: 7.56, Post: 8.53; t(33) = -2.47, p = .05; Cohen’s d = 0.33). The ability to focus attention was measured by the concentration ability index of the d2-R. Results showed significant improvements from pre (84.63) to post training (93.48; t(26) = -7.11, p < .001; Cohen’s d = 0.85). Finally, the improvement of reading abilities, as measured by the Alouette test, was significant on reading precision (z-score, Pre: -1.74, Post: -1.37; Wilcoxon: Z = 1.96, p = .05; Cohen’s d = 0.19) but not on reading speed). No pre to post improvements were found for the tests of Auditory Attention, Response Set, Digit Span and Visuomotor Precision.

### Descriptive analyses

In order to better understand the impact of the Démos music program at the individual level, each child was assigned to one of three groups, “increase”, “equal” or “decrease”, according to her/his pre-post-test evolution at the three main tests showing significant effects (total IQ, d2-R and Alouette reading precision). Results showed that 37% of children improved in all three tests, 50% improved in two tests, and 13% improved in one test.

### Cluster analyses

**Fig 4:**
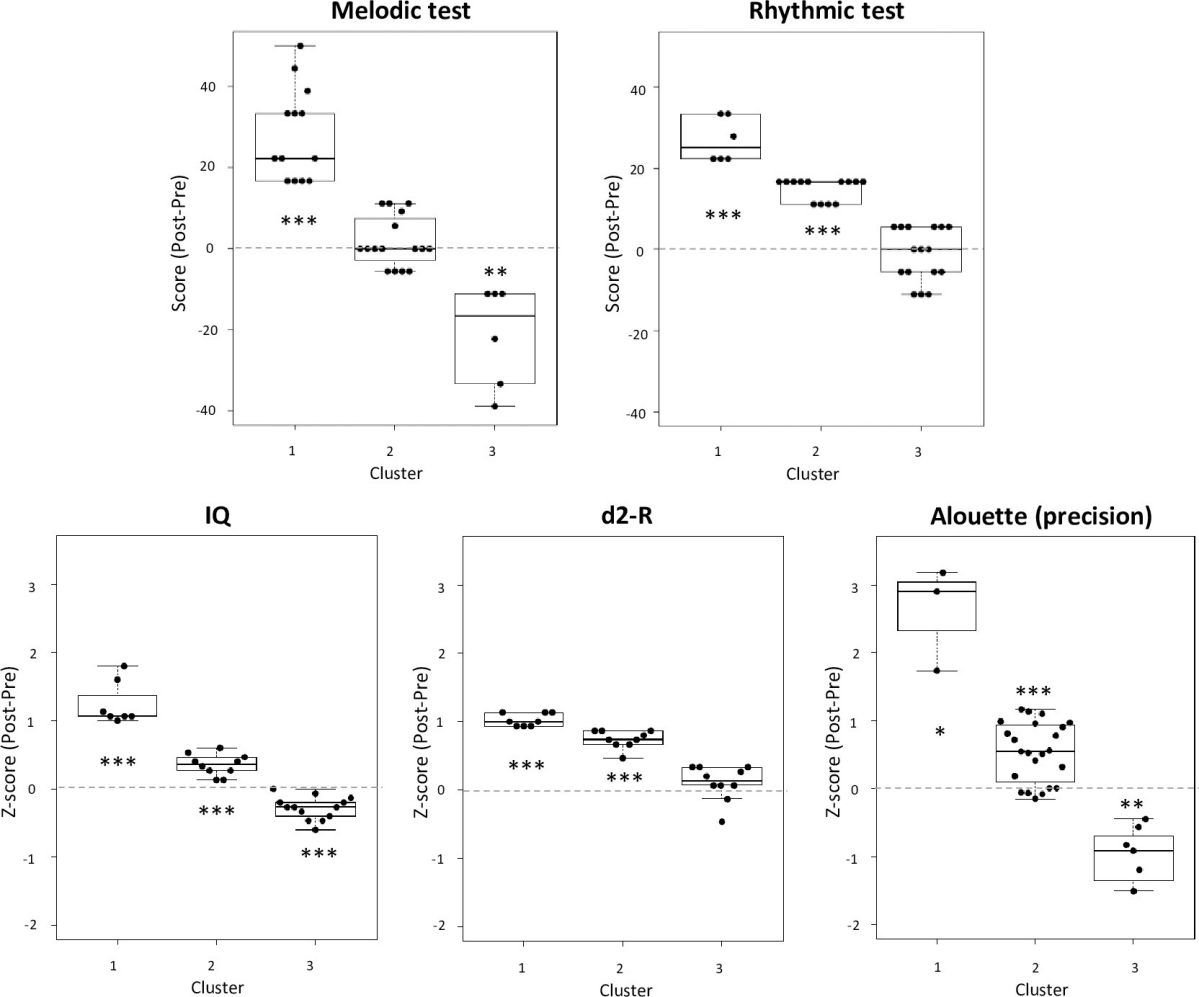
Results of cluster analyses for the five tests showing significant post minus pre- music training differences. For each test, each box plot corresponds to one cluster and the dots represent the children within each cluster. For each box plot, the upper quartile, the median and the lower quartile are represented together with the upper and lower whiskers. Significant increases or decreases in post minus pre-differences compared to 0 (no change) are indicated with *: p < .05; **: p < .01 and ***: p < .001.

Statistical results of cluster analyses are presented in Table 2 and illustrated on Fig 4. Except for the melodic and rhythmic tests (for which norms did not exist for our age groups), cluster analyses were performed on z-scores for the tests showing significant pre to post improvement (Rhythmic, Melodic, IQ, d2-R, Alouette). Results of the ANOVAs including Cluster as a within-subject factors confirmed the presence of three clusters that significantly differed from one another. To simplify results presentation, results are summarized in the text, presented in detail in Table 2 and illustrated on Fig 3. For the melodic test, the first cluster included 13 children (37%) showing a large significant improvement in melodic scores, the second included 16 children (46%) showing no significant improvement, and the third cluster included 6 children (17%) with a significant decrease in performance. In the rhythmic test, the first cluster included 6 children (17%) showing a large significant improvement in rhythmic scores, the second included 13 children (37%) with medium significant improvement, and the third cluster included 16 children (46%) with no significant improvement. Regarding IQ scores, the first cluster included 7 children (23%) with a large significant improvement in IQ scores, the second included 10 children (33%) with a medium significant improvement, and the third cluster included 13 children (43%) with a medium significant decrease in IQ scores. In the d2-R test, the first cluster included 8 children (30%) showing a large significant improvement in d2-R scores, the second included 9 children (33%) with medium significant improvement, and the third cluster included 10 children (37%) with no significant improvement. Finally, for the reading precision scores, the first cluster included 3 children (9%) showing a large significant increase in reading precision, the second included a larger group of 23 children (70%) with a small significant increase in reading precision and the third cluster included seven children (21%) with a small significant decrease in reading precision.

**Table 2:** Results of clusters analyses.

Test	Analysis of variance	Tukey HSD		Cluster 1	Cluster 2	Cluster 3	Inter-cluster Euclidian distances	
***Melodic-test***	F(2,32) = 61.15, p < .001	Always < .001	*N*	13 (37%)	16 (46%)	6 (17%)	*1–2*	26.59
			*Mean*	28.20	1.61	-21.30	*2–3*	22.90
			*Comparison to 0*	t(12) = 8.89, p < .001	t(15) = 1.05, p>.30	t(5) = -4.21, p < .01	*1–3*	49.50
***Rhythmic-test***	F(2,32) = 74.14, p < .001	Always < .001	*N*	6 (17%)	13 (37%)	16 (46%)	*1–2*	11.89
			*Mean*	26.85	14.96	-1.39	*2–3*	16.35
			*Comparison to 0*	t(5) = 12.04, p < .001	t(12) = 20.21, p < .001	t(15) = -0.85, p>.40	*1–3*	28.24
***IQ***	F(2,27) = 123.34, p < .001	Always < .001	*N*	7 (23%)	10 (33%)	13 (43%)	*1–2*	0.89
			*Mean*	1.25	0.35	-0.28	*2–3*	0.64
			*Comparison to 0*	t(6) = 10.42, p < .001	t(9) = 7.10, p < .001	t(12) = -5.97, p < .001	*1–3*	1.53
***d2-R***	F(2,24) = 62.50, p < .001	Always < .01	*N*	8 (30%)	9 (33%)	10 (37%)	*1–2*	0.28
			*Mean*	1.03	0.74	0.11	*2–3*	0.63
			*Comparison to 0*	t(7) = 30.89, p < .001	t(8) = 16.96, p < .001	t(9) = 1.33, p>.20	*1–3*	0.92
***Alouette (precision)***	F(2,30) = 57.56, p < .001	Always < .001	*N*	3 (9%)	23 (70%)	7 (21%)	*1–2*	2.07
			*Mean*	2.60	0.53	-1.12	*2–3*	1.66
			*Comparison to 0*	t(2) = 5.89, p < .02	t(22) = 5.80, p < .001	t(6) = -4.43, p < .01	*1–3*	3.73

### Regression analyses

**Fig 5:**
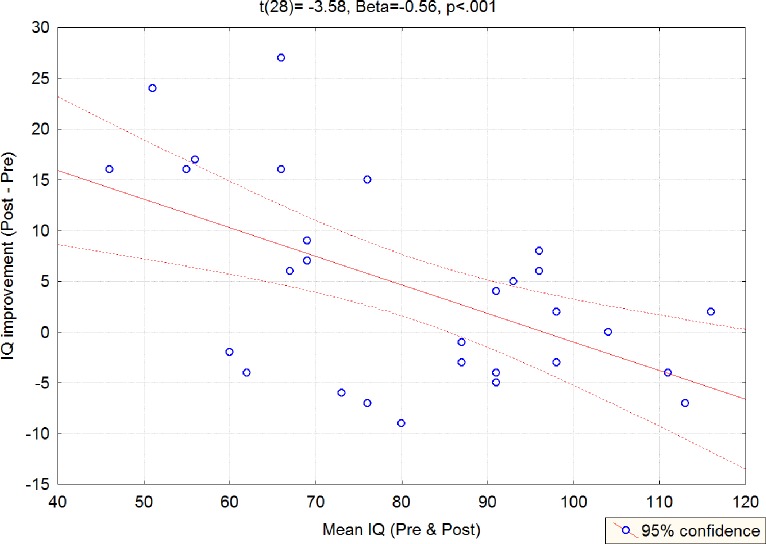
Results of simple regression analysis showing a significant correlation (p < .001) between initial IQ score and improvement in IQ.

Results of simple regression analyses to model the significant effects reported above and in particular, to determine whether improvements in IQ or in d2-R accounted for improvement in reading precision, as well as results of multiple linear regression analyses computed to model reading precision as a function of both IQ and d2-R showed no significant effects (effect of IQ on reading precision: t(27) = 0.22, Beta = .04, p = .82; effect of d2-R on reading precision : t(25) = -1.38, Beta = -0.27, p = .18). Interestingly, however, the improvement in IQ was significantly larger for children with initial lower IQ scores (t(28) = -3.58, Beta = -0.56, p < .001; see Fig 5).

Finally, results of multiple linear regression analysis, with IQ improvement (post minus pre) as the dependent variable and improvements in all four subtests as independent variables showed that all four subtests contributed to the improvement in IQ (Symbol Search: t(25) = 51.20, Beta = 0.44, p < .001; Similarities: t(25) = 56.19, Beta = 0.49, p < .001; Matrix Reasoning: t(25) = 59.20, Beta = 0.50, p < .001; Letter Number Sequencing: t(25) = 64.03, Beta = 0.55, p < .001).

## Discussion

Evaluation of the impact of the Démos program, initiated by the Paris Philharmonie and conducted in ecologically valid school-settings, on the cognitive development of children from low socio-economic backgrounds revealed several findings of interest after 18 months of music training. Results showed significant improvements of musicality scores, general intelligence (total IQ), processing speed (Symbol search) and concentration ability (d2-R) as well as increased reading precision (Alouette test). Results at the other tests were not significant although a trend in the same direction was observed (see Table 1).

### Musicality scores

The Démos music intervention was successful in improving the children’s level of performance in both the melodic and the rhythmic tests. In both cases, the effect sizes were relatively large (Cohen’s d = 0.55 and d = 0.70 respectively) and results of cluster analyses showed that more than half of children (54%) improved their abilities to judge whether two short musical phrases had identical or different rhythm. By contrast, only 37% of the children improved their ability to decide whether two musical phrases had identical or different melodic contour and 63% showed no improvement or a small decrease in melodic scores. Thus, while most children developed a better sense of rhythm, 18 months of music training were possibly not long enough to develop a “musical ear” for melody in most children. These results are in line with results from children of the middle class [42] showing that the melodic task is typically more difficult than the rhythmic task.

### General intelligence, processing speed and concentration abilities

In line with previous findings with typically developing children [1,2,4], one of the main finding of this study was a significant improvement of general intelligence (IQ scores) in children from low SES after 18 month of music training. This finding is of primary importance when considering that these children typically show lower IQ scores than children from higher socio-economic backgrounds ([31] for a review) and that this gap tends to increase over the course of development, the so-called “Matthew effect” [16,43]. For instance, Shaywitz & Shaywitz [17] followed-up a cohort of children for 7 years, from kindergarten to 6^th^ grade. IQ scores were evaluated every two years and, as in the present study, normalized-age scores were computed to rule out maturation and developmental effects. While children with higher IQ scores (e.g. average = 110) showed an increase in IQ, children with lower IQ scores (e.g. average = 80) showed a decrease in IQ over the course of elementary school. The children involved in the Démos program showed relatively low initial IQ scores (i.e., average = 80) and a small (effect size, Cohen’s d = 0.24) but significant increase in IQ of 4.3 points on average, that was close to the increase found in the control group of the Schellenberg’ study ([4]; mean IQ increase = 3.9). These results strongly suggest that the Démos music intervention program helped children from low SES to counteract the potential decrease in IQ over the school years [17] to get closer to the level of more privileged children. Future studies will aim at testing whether other types of training (e.g., dance, theater, painting …) can also counteract the decrease in IQ scores often encountered in children from low socio-economic backgrounds [17].

Results of cluster analyses are important in showing moderate (33% of the children) to strong (23% of the children) improvements in IQ scores after music training in more than half of the children (56%) involved in the Démos program compared to 43% showing a moderate decrease in IQ scores. While these increase and decrease in IQ may seem surprising, we need to keep in mind that they are computed on normalized scores that is, in reference to the results of a large children population within an age group. This population is, by definition, representative of the entire population and does not only include children from low SES. Thus, the decrease in IQ for children with low initial IQ scores, for instance, is relative to the entire population and may not be found if compared to specific norms for children from low SES. Moreover, the IQ increase was unlikely to be driven only by the significant improvement in the Symbols Search task since results of multiple regression analyses showed that the improvements in all four subtests contributed to the increase in IQ scores. Final but not least, results were very encouraging in revealing that children with lower initial IQ scores showed larger increases in IQ than children with higher initial scores. In other words, the impact of music training is potentially stronger when there is more space for improvement. These results are in line with recent results by Linnavalli et al. [19] showing that 5 to 6-year-old children with low scores in linguistic tasks benefitted more from music playschool activities than children who started with higher scores. They are also reminiscent of findings by Swaminathan and Schellenberg [44] showing that the impact of music training on musical competence (defined as the ability to perceive and remember sequences of tones or beats) was only significant in undergraduate (19-year old on average) who scored below the mean on tests of working memory and non-verbal intelligence. In sum, a tentative conclusion that needs to be tested in future studies is that individuals who initially scored relatively low on different tests may benefit more (as seen in the improvements of musical competence, linguistic tasks or IQ scores) from the positive influence of music training.

Based on previous results from cross-sectional studies in adults showing that musicians outperformed non-musicians in tasks similar to the Symbol Search task and the d2-R, taken to measure processing speed and concentration abilities [23], we expected children to improve at these tests after 18 months of music training. In line with these predictions, results showed significant improvements, with medium to large effect sizes in the Symbol Search (Cohen’s d = 0.33) and d2-R tests (Cohen’s d = 0.85). These findings provide further support to the proposal that playing music improves the ability to rapidly process information and to focus on the stimuli and task at hand. In this respect, it is remarkable that music training had a particularly strong impact (Cohen’s d = 0.85) on the concentration ability of a large percentage (63%) of children (as mentioned above, this increase is relative to the general child population in this age group and not compared to specific norms for children from low SES), possibly because they learned to focus attention on the teacher and on their instrument during the music classes. This finding is particularly interesting when considering that children from low SES often encounter difficulties to focus attention [28]. Along these lines, Neville et al. [29] showed that a family-based training program, targeting improvements of family stress regulation, parental responsiveness…, and of child attention, succeeded in improving selective attention in children from low SES. Thus, different types of training seem to exert a positive influence on attentional abilities. Moreover, by showing rapid improvement of selective attention (within 8 weeks in the Neville et al.’ intervention study) and of concentration abilities (within 18 months in the present study), these findings open new perspectives for research and education. While a few studies have tested the influence of musicianship on the variability of auditory cortical activity in adults [45] and in children [30], the impact of music training (longitudinal studies) on the different components of attention (e.g., selective vs divided attention, focused vs switching attention, distractors etc.) at the behavioral and electrophysiological levels has not yet been thoroughly examined. This is of primary importance for future research since the ability to focus attention on the task at hand is a prerequisite for successful learning in many, if not all, domains of knowledge. Future studies could test whether improvements in concentration ability associated to music training positively influence learning in other domains such as language or mathematics and whether these effects are specific to music training or could be found with other training activities.

### Phonological awareness, reading abilities and working memory

In line with results from the Kraus group with children from low SES musically-trained for 24 months within the Harmony program [14,15], results with children from the Démos program musically-trained for 18 months showed improvements in reading precision (Alouette test). Overall, these reading improvements were relatively small (Cohen’s d = 0.19) in most children (70%) but 9% of the children showed a large improvement and 21% showed a small decrease in reading precision, as shown by results of cluster analyses. Surprisingly, but in line with results from the Kraus group, we found no influence of music training on phonological awareness (First Phonemes Fusion and Syllabic Suppression). While it is very encouraging that similar results were found in two independent and culturally-different samples of children from low SES involved in music training, these results stand in contrast with the findings of Linnavalli et al. [19] and Nan et al. [20] showing a positive impact of playschool music and piano training on phonemic awareness and word discrimination based on consonants. However, in these two studies, children were younger (4-6-year old), they were not only issued from disadvantaged backgrounds and the phonological tests were different from the standardized tests used here, which possibly account for these differences. Nevertheless, our results were also unexpected based on results of meta-analyses pointing to stronger effects of music training on phonological awareness than on reading abilities [11,13] and in view of several findings showing that phonological abilities critically influence the development of reading skills [9].

Which factors contribute most to reading success is still a hotly debated issue [7]. For instance, recent results from a large sample of French children (N = 703) from low SES families [6] highlighted the primary importance of listening comprehension on the earliest phases of reading acquisition. Thus, lexical and semantic knowledge (e.g., what the words mean) seem to strongly influence reading abilities, while other factors, such as vocabulary, morphological and phonemic awareness may only indirectly contribute to reading skills (via listening comprehension). More generally, working and short-term memory [46], attention and executive functions [3,15,30,47,48], as well as articulatory rehearsal strategies [49] likely contribute to reading abilities. For instance, children with reading disabilities (dyslexics or poor readers) often show poor short-term phonological memory as revealed by scores at the forward digit span test and at the nonword repetition task (see meta-analysis [50]). Directly related to this issue, results of a longitudinal study by Perez and collaborators [51] showed that short term memory capacity predicted reading abilities one year later. These authors proposed that the ability to read new words is causally impacted by the ability to recall sequences of phonemes in the right order (taken to reflect core short-term memory [46]), rather than by phonological skills.

These views open the intriguing possibility that the positive influence of music training on reading precision reported here was mediated by improvements in general intelligence, concentration abilities, working and short-term memory rather than only by phonological skills. For instance, musical practice may improve concentration abilities on visual stimuli that, in turn, may facilitate grapheme-phoneme decoding and thereby improve reading performance. However, results of simple and multiple regression analyses showed that improvement in IQ scores and/or in concentration ability did not account for the improvement in reading precision. It may be that the effects size or the participant sample size were too small, with not enough children showing a large effect. Clearly more studies are needed to determine the relative weight of the different factors that contribute to the development of reading skills.

### Limitations

One could consider that the main limitation of the present study is the lack of an active control group which is needed to ascertain that the reported effects are specific to music training and would not obtain with another activity, such as dance, painting or cooking, that would be as interesting and as motivating for the children [4,5,52–54]. This, however, was not our aim and does not reduce the societal impact of the present findings. Whether effects similar to those obtained with the Démos music program were to be found with another type of training would also be very positive for the children. Moreover, since the pre-to-post comparisons reported here are based on normalized scores for each age group, we can ascertain that the results were not age-related, with children performing better because they were 18 months older post-than-pre training (i.e., we can rule out the influence of developmental and maturation effects). In fact, computing normalized scores is equivalent to comparing results of children trained with the Démos program to a passive control group that would include a large number of children representative of the population (e.g. 1103 children were tested for the French version of WISC-IV [33]) who are not involved in any specific training or who are waiting to be involved in music training, as in some previous studies [15,55]). .

A second potential limitation is linked to the relative high number of children who could not be tested post-music training because they left primary school to go to middle school or to another school. This is often the case in longitudinal studies and particularly when testing children from low SES. However, since only one child decided to stop music training, we are confident that the effects that we observed do not only come from children who were highly motivated by the Démos program compared to children that were less motivated.

A third limitation is linked to the repetition of the different tests with potentially higher level of performance on second than on first presentation. We partly controlled for this aspect by using tests that are typically chosen in the literature to evaluate the effects of an intervention. For instance, in a recent study [56] (see Table 3), children performed the French version of the WISC-IV twice, with an average of 20 months between test repetition. The improvement in IQ scores (Full Scale IQ) was significant but smaller (2.5) than in the present study (4.3). In the study by Ryan and collaborators [57], pre-post differences in IQ scores were not significant and smaller (1.6) than in our study, although the between-test repetition interval was shorter (11 months; see also [4,56,58]; Table 3). Finally, in the Shaywitz & Shaywitz’ study [17] discussed above, children with low initial IQ scores showed decreases, rather than increases, in IQ scores across the three repetitions of the same tests. It is thus unlikely that test repetition was driving the present results.

**Table 3:** Comparison of improvement in IQ scores across different experiments using test-re-test procedures with different repetition intervals.

*IQ*	Test–Retest interval; sample size	Protocol	ΔM	Cohen’s d
**Schellenberg, 2004** [4]	9 months; N = 132	Test—Music intervention (Keyboard)—Retest	6.1**	0.56
		Test—No training—Retest	3.9**	0.40
**Ryan et al., 2010** [57]	11 months; N = 43	Test—Retest	1.6	0.15
**Present study**	18 months; N = 35	Test—Music intervention—Retest	4.3*	0.24
**Kieng et al., 2017** [56]	19 months; N = 277	Test—Retest	2.5*	0.30
**Bartoi et al., 2015** [58]	20 months; N = 51	Test—Retest	-0.6	-

ΔM: mean difference in Retest minus Test scores; Cohen’s d: effect size.

*: p < .05 and

**: p < .01.

## Conclusions

Results of the present longitudinal experiment are important in showing that music training may counter-balance the negative influence of living in low socio-economic backgrounds [16] by enhancing several core cognitive functions: general intelligence, processing speed, concentration abilities and reading precision. Moreover, because these improvements were computed from normalized scores, it is possible to rule out the influence of maturation and developmental effects. However, as discussed above, we cannot ascertain that similar effects would not be found with another training program, as interesting for the children as the Démos music training program. But this is not problematic, on the contrary, as it would be important to use any training program that can counteract the negative effects of living in disadvantaged backgrounds. It is also important to note that several cognitive functions tested in the present study did not benefit from music training (auditory attention, visuomotor precision). For instance, in contrast to the results of Guo and colleagues [26] showing significant improvement in backward Digit Span scores after only 6 weeks of musical training, we did not find similar improvements of working memory. While the reasons for such variability in the results are difficult to determine, the question of whether music training has a general impact on cognitive abilities or whether its influence is specific to cognitive functions that share common processes with music, such as language for instance, is still an open issue [19,20,59–61]. In our view, the outcome most likely depends upon the duration of music training and upon the tasks used to test the effect of music training.

To conclude, and in agreement with the proposals made by several authors [14,61,62], we believe that ecological studies in school-settings, developed in partnership with existing programs and even if they are typically not as well controlled as laboratory-based experiments, are of crucial importance to better understand the impact of music training and of other types of training on the cognitive development of children from diverse cultural and economic backgrounds. Importantly, all children in the schools were from low SES and all were involved in the Démos music program: this reduced potential bias linked to children from high SES being more likely to choose music training. Moreover, children were strongly encouraged to practice their musical instruments. This is of crucial importance since motivation to pursue a demanding music training is likely to be lower in families facing social difficulties than in families with higher social status [19,63,64]. In sum, our results are encouraging in showing that, after only 18 months of music training, 37% of the children improved on three tests (general intelligence, concentration and reading abilities), and that 100% of the children improved at least in one of these tests. Thus, even if the effects were overall of small to medium size, as expected based upon the literature and the relatively short duration of music training, these positive results may help promote a wider use of music training in school-settings.
